# Syphilis in the Middle Ages and in Modern Times

**Published:** 1897-03

**Authors:** 


**Syphilis in the Middle Ages and in Modern Times.** By Dr. F. Buret. Translated by A. H. Ohmann-Dumesnil, M.D. Pp. xxvi., 289. Philadelphia: The F. A. Davis Company. 1895.

In September, 1892, we noticed Dr. Buret's *Syphilis in Ancient Times.* The book now before us is a continuation of that and completes the work, which, under the title of *Syphilis To-Day and Among the Ancients*, forms an exhaustive work which will prove of value to the syphilographer and medical historian; although there are many quotations from ancient documents which, we venture to think, most Englishmen would refrain from introducing into any publication, and which must render the work distasteful to many, however keen their pursuit after the proof of the early existence of syphilis.

The author shows conclusively that the plague of Naples in 1493 and 1494 was not an epidemic which burst suddenly upon the people of that city, but that it had been smouldering for several years before Charles VIII. marched into Italy and besieged Naples; that the French army, therefore, was not instrumental in introducing the disease directly, but that, by the overcrowding consequent upon the arrival of the troops, accompanied with hygiene of the worst character, a great and sudden increase in the epidemic was brought about. M. Buret has spent much time and exhibited no little patience in disproving the theory of "the American origin of syphilis," first promulgated by Schmaus in 1518 and perpetuated by the writings of Ulrich von Hutten. He does not deny the existence of the disease in America before the arrival of Columbus on its shores, but he proves up to the hilt that it was well-known in Italy years before.

The book closes with a few pages devoted to the prophylaxis of syphilis. A powerful protest is lodged against any coercive measures for its suppression.

